# Supplementary material for: Increasing ocean wave energy observed in Earth’s seismic wavefield since the late 20th century
Source: Nat Commun. 2023 Nov 1;14:6984. doi: 10.1038/s41467-023-42673-w (PMC10620394; doi:10.1038/s41467-023-42673-w)
Supplement: Supplementary file 1 — Supplementary Information [file 41467_2023_42673_MOESM1_ESM.pdf]

# Supplementary Information for: Increasing ocean wave energy observed in Earth's seismic wavefield since the late $20^{th}$ century

Richard C. Aster[1*], Adam T. Ringler[2], Robert E. Anthony[2] and Thomas A. Lee[3]

[1*]Geosciences Department, Warner College of Natural Resources, Colorado State University, 801 S. Howes St., Fort Collins, 80523-1482, Colorado, USA.
[2]Albuquerque Seismological Laboratory, U.S. Geological Survey, Target Rd. 10002 Isleta SE, Kirtland AFB, Albuquerque, 87117, New Mexico, USA.
[3]Department of Earth and Planetary Sciences, Harvard University, 20 Oxford St., Cambridge, 02138, Massachusetts, USA.

*Corresponding author(s). E-mail(s): rick.aster@colostate.edu; Contributing authors: aringler@usgs.gov; reanthony@usgs.gov; thomasandrewlee@g.harvard.edu;

. .

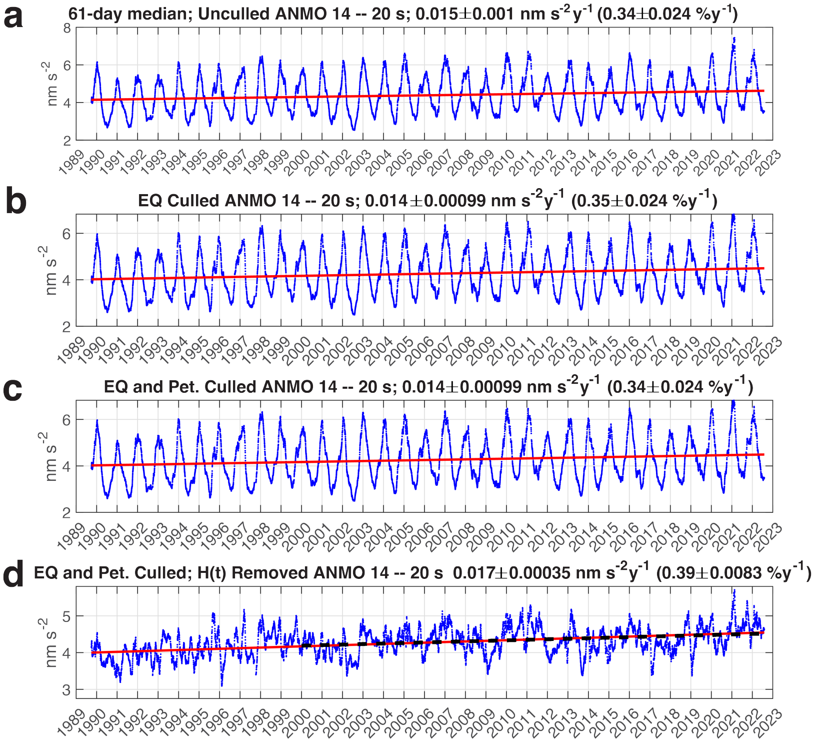


**Supplementary Fig. 1** Data characteristics and trend fitting example. Vertical component acceleration 14–20 s period microseism and $\ell_1$-norm fitting process (equation 6) for sequentially culled data at ANMO (Albuquerque, New Mexico). All data points are calculated from 50% overlapping hourly primary microseism velocity PSD integrals between 14 and 20 s smoothed with a two-month (61-day) median filter to daily samples. (a) All data; (b) Data culled using the ComCat earthquake catalog of all events with magnitudes M $\geq$ 5.75; (c) Earthquake-culled data (b) with out-of-noise corridor data estimated using [1] removed; d) the time series (c) with its associated stationary annual harmonic function (equation (1) subtracted. Note the robustness of absolute and percentage (relative to historical station median; Supplementary Fig. 4) estimates (red lines) throughout the various levels of processing as indicated in each subtitle. Confidence intervals in subtitles are $1\sigma$. Black dashed line in (d) shows the trend estimate for data since 1 January 2000.

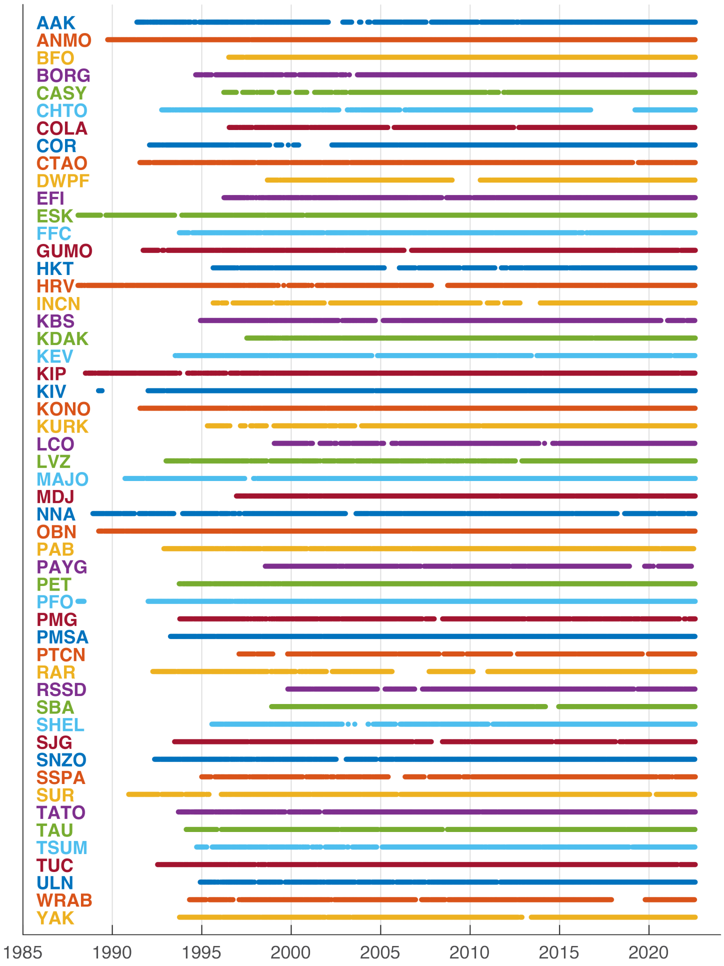


**Supplementary Fig. 2** Data intervals for all seismic stations used in this study. For details see Table 1 and Supplementary Table 1.

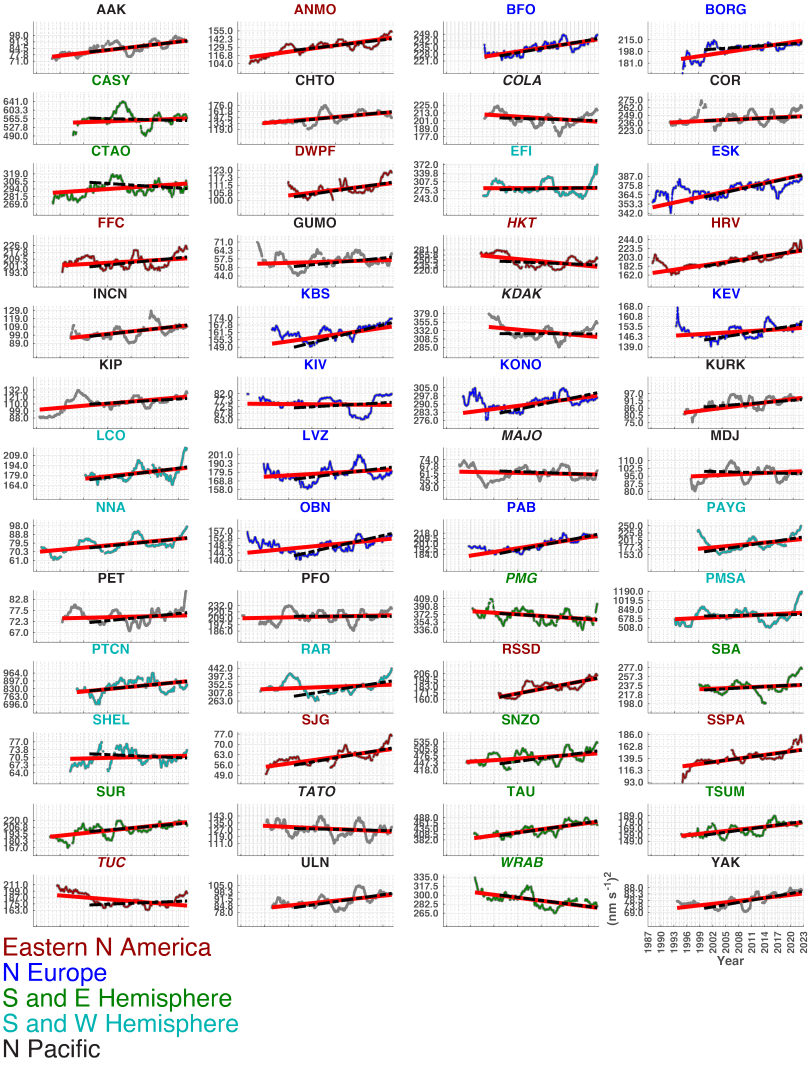


**Supplementary Fig. 3** Energy trends at all stations. $\ell_1$-norm minimizing trends (equation (6)) for the vertical component seismic velocity squared energy proxy (14–20 s period; Table 3) presented in the same format as for vertical component seismic acceleration in Fig. 1.

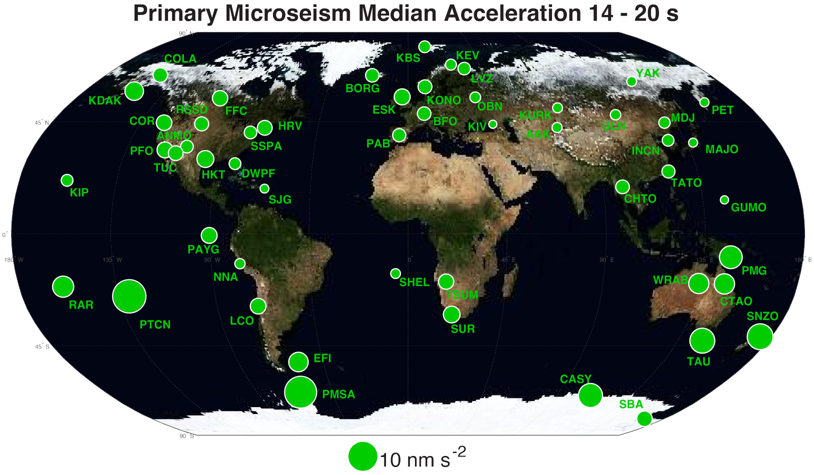


**Supplementary Fig. 4** Median primary microseism vertical component acceleration amplitudes for the operational history of each station through 1 August 2022.

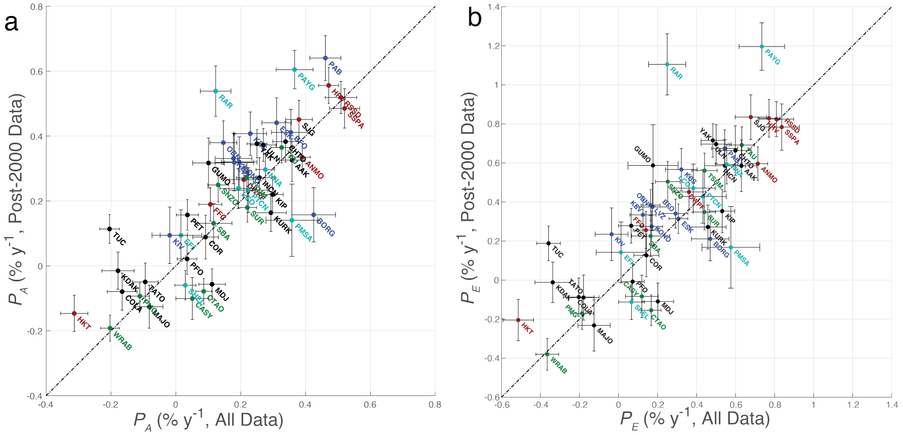


**Supplementary Fig. 5** All data versus post-2000 data trend comparisons. (a) Comparison of percentage trends for seismic acceleration for post-2000 data versus all data. (b) Comparative percentage trends for the seismic velocity squared energy proxy for post-2000 data versus all data. Dashed lines display 1:1 proportionality; stations above this line indicate an increased post-2000 trend and stations below a decreased post-2000 trend relative to trends for all data. Confidence intervals are $3\sigma$.)

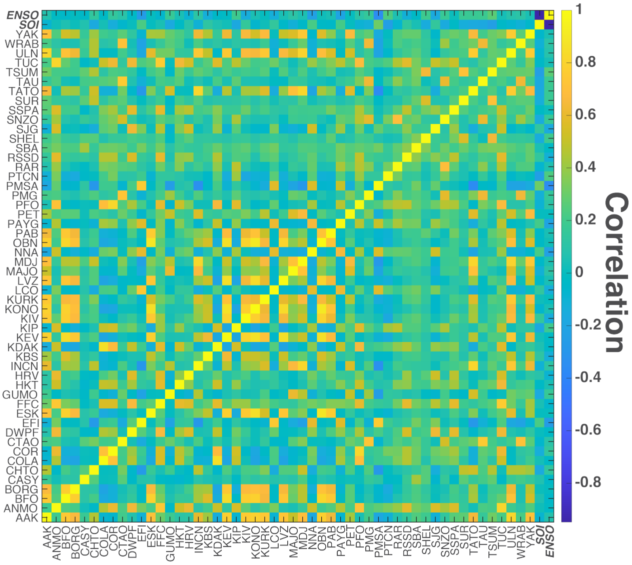


**Supplementary Fig. 6** Station time history correlation matrix. Correlation coefficients calculated from the detrended and demeaned 61-day smoothed time series of Fig. 6 used for clustering (Figs. 5, 7), including for the ENSO and SOI time series (Supplementary Fig. 7).

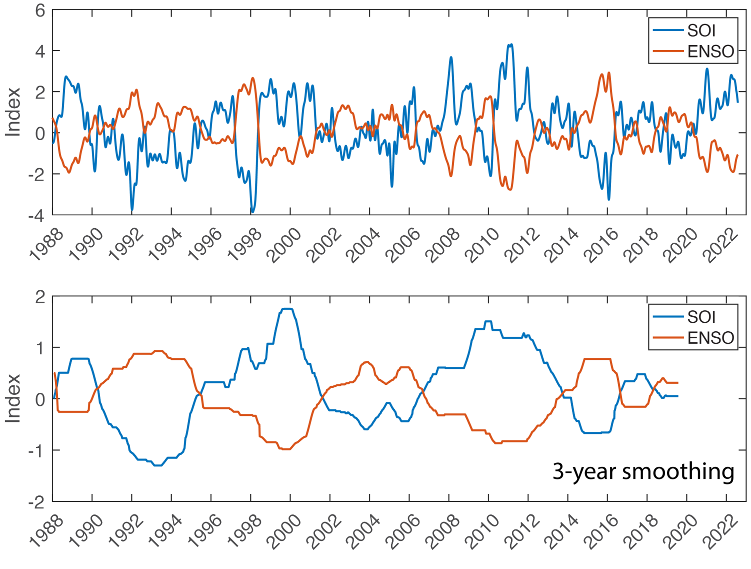


**Supplementary Fig. 7** Top: Southern Oscillation Index and Bivariate El Niño Southern Oscillation climate index time series, smoothed with a 61-day moving median window, used in clustering (Figs. 5, 7, Supplementary Fig. 6 [2, 3]). Bottom: Indices smoothed with a 3-year moving median window as shown in Fig. 7.

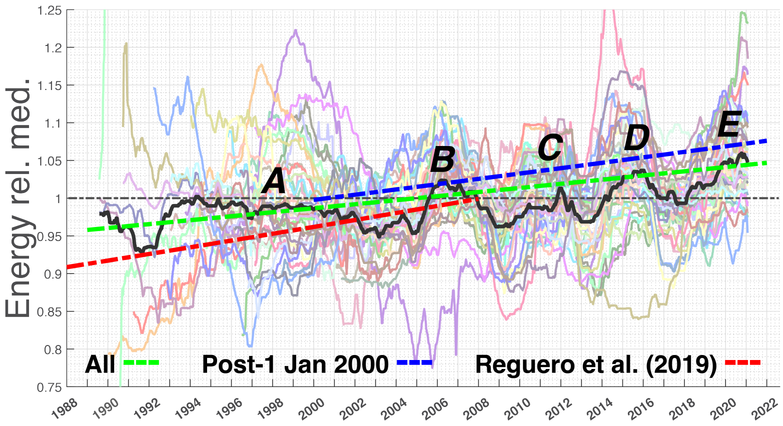


**Supplementary Fig. 8** Global seismic energy time series with the strongest ENSO index correlated (SWP and SEPSWA) stations removed. Note that ENSO-associated peaks $A$ – $E$ remain visible in the median (black) across all time series. Fig. 7b shows the same data presentation for all stations.

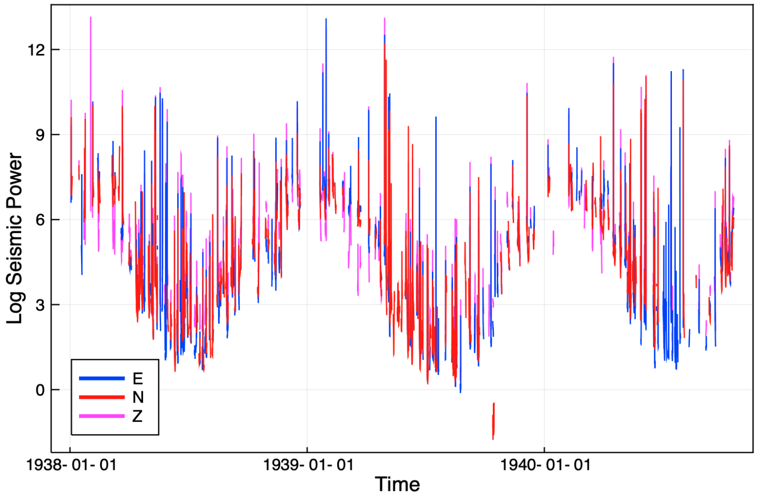


**Supplementary Fig. 9** Seismic power, with units derived from amplitude in pixels, of scanned and digitized long-period records for three seismic components at Harvard, Massachusetts (site of the present HRV network station) from the beginning of 1938 to the end of 1940. Note the resolution of seasonal three-component secondary microseism trends, showing characteristic elevated amplitudes during the northern hemisphere winter.

| Station Name | Start Date | End Date |
|---|---|---|
| CHTO | 1 Feb. 2006 | 1 May 2006 |
| CHTO | 15 Oct. 2016 | 1 Mar. 2019 |
| COLA | 1 Jun. 2005 | 1 Oct. 2005 |
| COR | 1 Jul. 2000 | 1 Apr. 2002 |
| GUMO | 9 June 1991 | 22 Sep 1991 |
| GUMO | 3 May 2006 | 20 Sep 2006 |
| INCN | 1 Aug. 2010 | 15 Dec. 2010 |
| LCO | 1 Nov. 2014 | 15 Dec. 2014 |
| RSSD | 1 Dec. 2006 | 1 May 2007 |
| SJG | 1 Nov. 2007 | 7 Jun. 2008 |
| TATO | 26 Sep. 1992 | 1 Jul. 1993 |
| WRAB | 1 Nov. 1996 | 1 Feb. 1997 |

**Supplementary Table 1** Station names, start dates, and end dates for contiguous time intervals at ten stations for which corrupted data were removed following visual inspection of 50% overlapping hourly PSD integral time series. These intervals are reflected as gaps along with general station operational outages in Supplementary Fig. 2.

# References

[1] Peterson, J.: Observations and modeling of seismic background noise. U.S. Geological Survey Open-File Report 93-322, 94 (1993)

[2] Ropelewski, C.F., Halpert, M.S.: Global and regional scale precipitation patterns associated with the El Niño/Southern Oscillation. Monthly Weather Review **115**(8), 1606–1626 (1987). https://doi.org/10.1175/1520-0493(1987)115⟨1606:GARSPP⟩2.0.CO;2

[3] Smith, C.A., Sardeshmukh, P.D.: The effect of ENSO on the intraseasonal variance of surface temperatures in winter. International Journal of Climatology **20**(13), 1543–1557 (2000). https://doi.org/10.1002/1097-0088(20001115)20:13⟨1543::Aid-joc579⟩3.0.Co;2-a
